# Supplementary material for: Resilient performance in healthcare during the COVID-19 pandemic (ResCOV): study protocol for a multilevel grounded theory study on adaptations, working conditions, ethics and patient safety
Source: BMJ Open. 2021 Dec 7;11(12):e051928. doi: 10.1136/bmjopen-2021-051928 (PMC8655346; doi:10.1136/bmjopen-2021-051928)
Supplement: Author's manuscript [file bmjopen-2021-051928.draft_revisions.pdf]

BMJ Open

BMJ Open is committed to open peer review. As part of this commitment we make the peer review history of every article we publish publicly available.

When an article is published we post the peer reviewers' comments and the authors' responses online. We also post the versions of the paper that were used during peer review. These are the versions that the peer review comments apply to.

The versions of the paper that follow are the versions that were submitted during the peer review process. They are not the versions of record or the final published versions. They should not be cited or distributed as the published version of this manuscript.

BMJ Open is an open access journal and the full, final, typeset and author-corrected version of record of the manuscript is available on our site with no access controls, subscription charges or pay-per-view fees (http://bmjopen.bmj.com).

If you have any questions on BMJ Open's open peer review process please email info.bmjopen@bmj.com

Resilient performance in healthcare during the COVID-19 pandemic (ResCOV): study protocol for a multilevel grounded theory study on adaptations, working conditions, ethics and patient safety.

Journal:	*BMJ Open*
Manuscript ID	bmjopen-2021-051928
Article Type:	Protocol
Date Submitted by the Author:	01-Apr-2021
Complete List of Authors:	Bjurling-Sjöberg, Petronella; Uppsala University, Department of Public Health and Caring Sciences, Caring Science; Region Sörmland, Centre for Clinical Research, Uppsala University<br>Göras, Camilla; Region Dalarna, Department of Anesthesia and Intensive Care Unit, Falun Hospital; Dalarna University School of Education Health and Social Studies, School of Education, Health and Social Studies<br>Lohela-Karlsson, Malin; Region Västmanland, Centre for Clinical Research, Uppsala University; Uppsala Universitet, Department of Public Health and Caring Sciences<br>Nordgren, Lena; Uppsala University, Department of Public Health and Caring Sciences, Caring Science; Region Sörmland, Centre for Clinical Research Sörmland, Uppsala University<br>Källberg, Ann-Sofie; Dalarna University School of Education Health and Social Studies, School of Education, Health and Social Studies; Region Dalarna, Department of Emergency Medicine, Falun Hospital<br>Castegren, Markus; Karolinska Institute, CLINTEC; Region Sörmland, Centre for Clinical Research, Uppsala University<br>Condén Mellgren, Emelie; Region Västmanland, Centre for Clinical Research, Uppsala University<br>Holmberg, Mats; Region Sormland, Centre for Clinical Research Sörmland, Uppsala University; Linneuniversitet, Department of Health and Caring Sciences<br>Ekstedt, Mirjam; Linneuniversitet, Department of Health and Caring Sciences; Karolinska Institutet, LIME
Keywords:	COVID-19, ETHICS (see Medical Ethics), Health & safety < HEALTH SERVICES ADMINISTRATION & MANAGEMENT, QUALITATIVE RESEARCH, Human resource management < HEALTH SERVICES ADMINISTRATION & MANAGEMENT, Quality in health care < HEALTH SERVICES ADMINISTRATION & MANAGEMENT

I, the Submitting Author has the right to grant and does grant on behalf of all authors of the Work (as defined in the below author licence), an exclusive licence and/or a non-exclusive licence for contributions from authors who are: i) UK Crown employees; ii) where BMJ has agreed a CC-BY licence shall apply, and/or iii) in accordance with the terms applicable for US Federal Government officers or employees acting as part of their official duties; on a worldwide, perpetual, irrevocable, royalty-free basis to BMJ Publishing Group Ltd ("BMJ") its licensees and where the relevant Journal is co-owned by BMJ to the co-owners of the Journal, to publish the Work in this journal and any other BMJ products and to exploit all rights, as set out in our licence.

The Submitting Author accepts and understands that any supply made under these terms is made by BMJ to the Submitting Author unless you are acting as an employee on behalf of your employer or a postgraduate student of an affiliated institution which is paying any applicable article publishing charge ("APC") for Open Access articles. Where the Submitting Author wishes to make the Work available on an Open Access basis (and intends to pay the relevant APC), the terms of reuse of such Open Access shall be governed by a Creative Commons licence – details of these licences and which Creative Commons licence will apply to this Work are set out in our licence referred to above.

Other than as permitted in any relevant BMJ Author's Self Archiving Policies, I confirm this Work has not been accepted for publication elsewhere, is not being considered for publication elsewhere and does not duplicate material already published. I confirm all authors consent to publication of this Work and authorise the granting of this licence.

For peer review only

TITLE

Resilient performance in healthcare during the COVID-19 pandemic (ResCOV): study protocol for a multilevel grounded theory study on adaptations, working conditions, ethics and patient safety.

Authors

Petronella Bjurling-Sjöberg, CCRN, MSc, PhD [a,b*]

Camilla Göras, RNA, MSc, PhD [c,d]

Malin Lohela Karlsson, PhD [e,a]

Lena Nordgren, RN, MSc, PhD [a,b]

Ann-Sofie Källberg, RN, MSc, PhD [d,f]

Markus Castegren, MD, PhD [g,b]

Emelie Condén Mellgren, RN, PhD [e]

Mats Holmberg, RN, PEN, PhD [b,h]

Mirjam Ekstedt, RN, PhD [h,i]

Author affiliations

[a] Department of Public Health and Caring Sciences, Caring Science, Uppsala University, Uppsala, Sweden

[b] Centre for Clinical Research Sörmland, Uppsala University, Eskilstuna, Sweden

[c] Department of Anesthesia and Intensive Care, Falun Hospital, Region Dalarna, Falun, Sweden

[d] School of Education, Health and Social Studies, Dalarna University, Falun, Sweden

[e] Centre for Clinical Research, Region Västmanland, Uppsala University, Västerås, Sweden

[f] Department of Emergency Medicine, Falun Hospital, Falun, Sweden

[g] CLINTEC, Karolinska Institute, Stockholm, Sweden

For peer review only

[h] Department of Health and Caring Sciences, Linnaeus University, Kalmar/Växjö Sweden

[i] Department of Learning, Informatics, Management and Ethics, Karolinska Institute, Stockholm, Sweden.

*Corresponding Author. Postal address: Department of Patient safety, Mälar Hospital, 631 88 Eskilstuna, Sweden; Telephone: +46 76 885 75 92; email address: Petronella.bjurling-sjoberg@pubcare.uu.se

For peer review only

ABSTRACT

Introduction

Since early 2020, the COVID-19 pandemic has challenged societies and revealed the built-in fragility and dependencies in complex adaptive systems, such as healthcare. The pandemic has placed healthcare providers and systems under unprecedented amounts of strain with potential consequences that have not yet been fully elucidated. This multilevel project aims to explore resilient performance with the purpose of improving the understanding of how healthcare has adapted during the pandemic's rampage, the processes involved, and the consequences on working conditions, ethics and patient safety.

Methods

An emerging explorative multilevel design based on grounded theory methodology is applied. Open and theoretical sampling is performed. Empirical data are gathered from throughout the pandemic from written narratives and qualitative interviews with staff with different professions and positions in healthcare organizations in two Swedish regions with seven hospitals and outpatient care. The participants' first-person stories are complemented with data from the healthcare organizations' internal documents and national and international official documents.

Analysis

Experiences during the pandemic and expressions of resilient performance at different system levels and times, existing influencing risk and success factors at the micro, meso, and macro levels and interrelationships and consequences in different healthcare contexts, are explored using constant comparative analysis. Finally, the empirical data are complemented with the

For peer review only

current literature to develop a substantive theory of resilient performance during the pandemic.

Ethics and dissemination

This project is ethically approved and recognizes the ongoing strain on the healthcare system when gathering data. The ongoing pandemic provides unique possibilities to study system-wide adaptive capacity across different system levels and times, which can create an important basis for designing interventions focusing on preparedness to manage current and future challenges in healthcare.

Registration

The project is preregistered in The Open Science Framework (http://osf.io DOI 10.17605/OSF.IO/Y3P78).

Keywords

Resilience, COVID-19, Grounded theory, Healthcare, Patient safety, Ethics, Worker health, Moral distress

For peer review only

Strengths and limitations of this study

- The first project that uses grounded theory to study working conditions, ethics and patient safety during a healthcare crisis through a lens of resilience.
- Using an exploratory multilevel design with openness for the main concern of the participants can generate in-depth knowledge to understand better the nature and context of adaptations, the interplay between different system levels, the success factors for resilient performance, and the influencing factors and consequences.
- Empirical data are gathered over time from healthcare organizations in two mid-size Swedish regions with seven hospitals and outpatient care, and the transferability of the findings is enhanced by contextual descriptions.
- The project enables the development of theoretical understanding from empirical data, and the findings can create an important basis for designing interventions with a focus on preparedness to manage current and future crises and challenges in healthcare.
- The project is heavily affected by the progress of the COVID-19 pandemic and the staff's ability to participate; therefore, the research trajectory is difficult to predict precisely.

For peer review only

INTRODUCTION

Since early 2020, the COVID-19 pandemic has challenged individuals, healthcare systems, and governments, which has revealed the built-in fragility and dependencies in the complex system that constitute current societies.[1] The response to the threat has varied among nations, but overall, the gravity of the situation was initially underestimated.[2-4] In Sweden, the first COVID-19 cases, without connection to travelers, were diagnosed in early March 2020.[5] Calculations regarding progress, including potential effects on healthcare, were soon surpassed as the pandemic hit some regions substantially earlier and harder than predicted, and the need for hospital care increased exponentially. Hence, healthcare organizations rapidly had to escalate the capacity of care to an unprecedented extent. This factor, together with initially limited knowledge about the disease and appropriate treatment, created ad hoc solutions and uncertainty at all levels. In July, the first wave of the pandemic ebbed out, and healthcare could return to relatively ordinary conditions. In autumn, a second wave hit the nation. Additionally, in the spring of 2021, extensive vaccination efforts with unstable vaccine deliveries were organized and manned. Over the past year, the pandemic has forced healthcare organizations and involved staff to be on alert and to frequently adapt to changing conditions. Additionally, at the time of writing, mutations of the virus created a third wave, contributing to further uncertainty about the future.

Although healthcare professionals around the world are used to encountering crises and events, the pandemic has forced them to work under extremely stressful conditions, which has challenged their performance and ability to provide safe care. The severity of the disease and the high in-flow of patients have also meant prioritizing and potentially ethical dilemmas and moral distress. In addition to ethical decisions and facing mortalities far beyond those of previous experience, additional related stressors may include patient overflow, additional

For peer review only

shifts, altered medical interventions, insufficient access to protective equipment and intervening outside the area of expertise.[6 7] Depressive symptoms, anxiety, psychological distress, poor sleep quality[8] and posttraumatic stress disorder[9] have been reported by healthcare professionals. Significant strain on the healthcare system entails an increased risk for diagnostic errors, adverse events and delayed care for patients with COVID-19 as well as other illnesses.[10 11 12] A Swedish medical record review[13] revealed that 18.1% of COVID-19 patients were affected by adverse events during March 2020 to June 2020. This level is twice as high as the frequency for other patients during the same period. Patients admitted to intensive care units were exposed to a four times higher risk of injury.[13] However, experiences and effects of the pandemic are still being elucidated, and knowledge of the processes involved in maintaining safe performance and what support healthcare professionals need in such mega-events is deficient.[14] To generate this additional knowledge, the complexity, interactions and dynamic adaptations that have been made in current societies and healthcare systems need to be considered.[15]

Complex adaptive systems, such as healthcare, are open and constantly interact with their environment. They also contain many elements at different levels that interact in dynamic processes over time.[1 16 17] The micro level of the system, where the patient´s urgent needs require rapid decisions, sometimes operates at different temporalities than the meso- and macro-levels, from which the system's resources and management are controlled and regulated. This issue challenges the ability to anticipate and to understand the larger view of the system and to learn from macro-outcomes in addition to the micro.[18] The ability of a complex adaptive system to self-organize makes it resilient to most disturbances, but changing dynamics also have the potential to destabilize the system.[1] To understand work and

For peer review only

successful responses to challenges and problems in complex adaptive systems, resilience and explicit resilient healthcare have received increased attention.[19 20]

Resilience requires that an organization have the 'potential' for resilient performance, i.e., the capacity to act in specific ways under certain conditions.[19] Resilience in healthcare is defined as 'the capacity to adapt to challenges and changes at different system levels, to maintain high-quality care'.[21] This perspective involves a proactive approach, which requires the potential to anticipate, to monitor, to learn and to respond.[19] However, resilience is not normative, as adaptations also have the potential for negative consequences.[20] Safety science warns that reducing resilient performance to individuals' capacity to face challenges and complexity may lead to the creation of safety strategies that rely primarily on individuals, and not on system safety.[22 23]

Today, patient safety is often assessed through analysis and the quantification of deviations. By accepting that healthcare is a complex adaptive system and adopting a system perspective with a focus on preventing and strengthening success, a more proactive approach emerges.[24] Such an approach may improve patient safety and quality of care, prevent worker ill health and achieve good working conditions. However, although there has been an increased focus on proactive safety work, success factors regarding working conditions and patient safety are still relatively unexplored.[24 25] Additionally, despite the importance of learning, success factors for adaptive capacity are relatively unexplored,[26] and knowledge about how resilient performance is created is still deficient.[14 15 24 27] Except for some notable ongoing studies,[28 29] empirical studies on resilience have focused mainly on the micro level.[30 31] To extend the understanding of resilient performance and to explain important interactions between different

For peer review only

system levels in healthcare, there is a need to contribute to the theory of resilient healthcare with empirical data from a multilevel perspective.[20 31]

The pandemic has placed healthcare providers and systems under unprecedented strain, with potential consequences on working conditions, ethics and patient safety that have not yet been fully elucidated. The adaptive capacity of a complex adaptive system under extreme pressure can never be fully understood. However, the ongoing pandemic provides unique possibilities to study system-wide adaptive capacity in real time. Applying a multilevel approach and studying how professionals at different levels of the system adapt their operations under the ongoing pandemic will increase the understanding of how adaptive processes interplay across care contexts and system levels to sustain high-quality performance and safety under extraordinary conditions. This approach will also increase knowledge about useful adaptations and success factors for how resilience is created through rapid decisions and adaptations at the micro level of the system and in the interplay between the micro- and meso-level. The staff´s experiences of ethical stress and workload will also be explored. Thus, empirical exploration of resilient performance, influencing factors, and consequences across different system levels and times can create an important basis for designing interventions focusing on preparedness to manage current and future challenges in healthcare.

Aims and objectives

This study protocol describes the research project 'Resilient performance in healthcare during the COVID-19 pandemic (ResCOV)'. The overall aim of this multilevel project is to improve the understanding of how healthcare adapted during the pandemic's rampage, the processes involved, and the consequences on working conditions, ethics and patient safety. The specific objectives are as follows:

For peer review only

I. To explore and describe how staff from different professions and positions in healthcare organizations experienced and managed the pandemic's rampage and thus influenced factors and consequences.

II. To explore and identify expressions of resilient performance at different system levels and times, influencing risk and success factors at the micro-meso-macro levels, the interrelationships and consequences on sustainable working conditions, and ethics and patient safety in different healthcare contexts.

III. To develop a substantive theory to provide a comprehensive contextualized explanation of resilient performance within healthcare during the pandemic.

METHODS AND ANALYSIS

Overall design

To explore resilient performance during the COVID-19 pandemic's rampage, this project has an emerging explorative multilevel design based on the grounded theory methodology[32 33] (see Figure 1). This type of design is ideal when the aim is to understand phenomena in a research area where previous knowledge is limited and where the concerns of people involved are in focus. The project started with data gathering in September 2020, which will continue until theoretical saturation is achieved, i.e., when sufficiently comprehensive data to thoroughly understand the characteristics of the phenomenon and process is obtained.[32 33] How long this process will take is greatly dependent on the progress of the pandemic. To address the overall aim and the three study objectives, the research will be conducted in four iterative, flexible and closely integrated cycles of activities, including staff from different professions and levels in healthcare organizations, complemented with data from different documents, as specified below.

For peer review only

Cycle I, micro level: To explore how healthcare professionals experienced and managed the pandemic's rampage, empirical data from professionals working with COVID-19 patients in different contexts, such as emergency departments, specific COVID units and intensive care, will be gathered. The datasets will be analyzed separately and focus on each profession at the time. Interactions with nurse managers and medical directors, as well as interprofessional interactions, influencing factors and consequences in different phases of the pandemic, will also be analyzed in the first cycle.

Cycle II, meso level: Empirical data from the staff in positions not directly involved in patient care, such as managers at the meso level and service departments etc., will be gathered and analyzed; the focus will be on their experiences, i.e., interorganizational interactions, decisions and adaptations, influencing factors and the consequences on micro- and meso-level performance at different phases of the pandemic.

Cycle III, multilevel (meso-macro) level: Empirical data from key informants in context indirectly affected by the pandemic, temporary disaster management of healthcare organizations, internal documents from healthcare organizations, and official national and international documents will be gathered and integrated with the findings from cycles I and II. These integrated data will be analyzed cross-sectionally and consider the different system levels and phases of the pandemic by focusing on resilient performance, the influencing factors and the consequences in each of the areas of specific interest, namely, sustainable working conditions, ethics and patient safety.

Cycle IV. Finally, we will develop a comprehensive contextualized explanation of resilient performance within healthcare during the pandemic. The findings from cycles I-III will be synthesized and supplemented with knowledge from the current literature to provide

For peer review only

an empirical and theoretical foundation for the development of a substantive theory of resilient performance during the pandemic rampage.

Please, insert Fig. 1

Study context

Swedish public healthcare provide the setting for the project. Healthcare in Sweden is largely publicly funded. National regulations provide a foundation for the healthcare system, but the management is self-governed by the 21 regional councils. Therefore, each region is responsible for the organization and prioritization of healthcare resources.[34] The clinical healthcare professionals mainly include registered nurses, some who are specialized within certain fields, e.g., intensive care and anesthesiology; undergraduate assistant nurses; physicians specialized within certain fields, such as medicine, surgery or anesthesiology, and those under training; and some allied healthcare professions, such as physiotherapists, occupational therapists, dieticians and counselors. Unlike some other countries, drugs and mechanical ventilation are handled within the healthcare team without pharmacists or respiratory therapists.

The project is performed in the healthcare organizations of two Swedish regions, including seven somatic hospitals and outpatient care. The regions each have a population of ~300 000, which is approximately the median size for the nation. The total number of in-hospital beds in each region is 500-600, with 70-300 beds/hospital, which is a common size of local or county hospitals in the nation.

Sample and Participants

For peer review only

Recruitment started September 2020 and is ongoing. Potential participants are contacted through the organizations' e-mail systems. They are informed about the study, aim, procedure and voluntariness and asked about participation. Initially, aiming to maximize variations in experiences in cycles I-III, open sampling[33] was conducted by recruiting staff from different professions, positions and contexts in healthcare organizations. First, healthcare professionals (registered nurses, assistant nurses, physicians, allied healthcare professions and clinical managers) were recruited who, on a regular or temporary basis, were on duty in the sectors of healthcare that were most affected by the first wave of the pandemic, including emergency departments, specific COVID units, intensive care and operating room departments. Second, higher-level managers were recruited from all parts of the hospitals and primary care, key informants from selected service and support departments, and top-level management in the healthcare organizations and temporary disaster management. At the time of this writing, March 2021, just over 200 participants have been included.

Later, guided by the simultaneously performed analysis, theoretical sampling[33] will take place, and data be gathered to explore differences over time, to fill gaps in understanding and to saturate emerging concepts and categories. In this phase, we will return to already recruited participants for complementary information and recruit additional participants if needed. Additionally, data will be utilized from internal documents from healthcare organizations, such as statistics, guidelines, protocols, and official national and international documents, including statistics, information, guidelines, recommendations, and legal regulations.

Data collection

Primary data consist of the participants´ first-person written or verbal narratives, which are supplemented with data from documents as specified above. Data collection started simultaneously with recruitment and will continue throughout the course of the pandemic until theoretical saturation[32 33] is achieved. Individuals who consent to participate are given the choice to tell their story in writing or to participate in individual qualitative interviews. They are also asked to fill out a questionnaire for some demographic data (gender, age, occupation and years in this occupation), regular workplace, role and duties, and workplace, role and duties during the pandemic. Additionally, participants are asked to attach supplementary documents from their workplace that they think could facilitate the researchers understanding of their story and to provide contact information if they consent to be contacted again for further questions.

The participants are encouraged to tell their story about their experiences, thoughts and feelings during the pandemic openly. To aid the written narrative, a study-specific guide has been created based on a review of publications and the researchers' experiences. The face validity of the guide was tested by six healthcare professionals, and after minor revisions it was assessed as valid. The guide includes some guiding questions for the topics of specific interest (working conditions, ethics, patient safety, adaptations, influencing factors, consequences and lessons learned) but urges the participants to tell or to leave out whatever they want.

The interviews are performed by the researchers with great openness for what the informant thinks is important to illuminate, starting with the question 'Can you please tell me how the situation been during the pandemic?' and continuing with illuminating probing questions. A semistructured interview guide has been developed, which includes the same topics as those

For peer review only

found in the guide for the written narratives; however, this guide is used in a flexible manner to ensure that the participants' concerns are captured. Based on the ongoing analysis, both guides are successively refined to support exploration of emerging understandings. All interviews are digitally recorded and transcribed verbatim.

Data collection from documents on different system levels and periods of time will be conducted first through the participants who provide internal documents from their workplace. Second, the researchers will request useful official documents from healthcare organizations and authorities or collect them from their websites if available.

Data management and analysis

Demographic data are managed in SPSS Statistics[35] to provide descriptive statistics of the participants. All qualitative data are managed in NVivo[36] and analyzed using constant comparative analysis according to the principles of grounded theory.[33] The analysis process started with the first data collection and will continue until theoretical saturation is achieved in all parts of the project.

Throughout the analysis process, data and emerging concepts and categories are compared back and forth, moving between open, axial and selective coding as new data are collected and the theorizing proceeds. The theorizing includes reflecting, writing memos and drawing diagrams of interpretations, ideas, assumed associations and theoretical reflections related to each emerging category, moving towards explanation in an abductive approach,[37] shifting between inductive interpretation and deductive testing as hypotheses evolve. Finally, the developed theory will be validated in the original empirical raw data.

For peer review only

The development of theoretical understanding is a process between the researcher and the empirical data, and the voice of the participants is rendered into the findings. The level of abstraction/theorization in the studies depends on the content and depth of the data obtained. Hence, the research team is open to settling with a lower level of abstraction, such as purely descriptive results or conceptual modeling in some studies. These results can also be of substantial usefulness. The most important quality criterion in grounded theory is that the findings are truly grounded in the data. The findings should 'fit' the empirical situations in the social context under study, 'work' to explain what was going on in the studied context and have 'relevance' by representing subjects of real concern to the people involved. Finally, the developed theory or conceptual model should have 'modifiability', meaning that as changes take place in reality and studies are performed in other contexts, the theory can evolve.[33 38]

Based on the philosophical orientation of the project, reality is multiple, complex, socially constructed, subjectively perceived, and can be interpreted but not fully known. A further assumption is that knowledge is created within the interaction between the researchers and the participants. Consequently, objectivity is not desirable. However, to ensure that the findings are grounded in the empirical data, the researchers have to be reflective regarding their own pre-understanding.[39] Strategies appropriate for qualitative studies are applied to achieve trustworthiness.[40] The primary analysis in each study will be conducted by two researchers, and reflective discussions will take place with other members of the research team. Clinical experiences within the research team enable theoretical sensibility. Credibility is enhanced through an audit trail, collaboration in the analysis, comprehensive memo writing, and reflective discussions within the research group, which include individuals without experience of care during the pandemic, an audit trail, peer reviews and in-depth methodological

For peer review only

descriptions. The transferability of the findings will be enhanced by in-depth contextual descriptions.

ETHICS AND DISSEMINATION

The project was approved by the Swedish Ethical Review Authority (Ref. No. 2020-04187). The continuing effect of the pandemic on healthcare means that the researchers have to be responsive to the participants' conditions, both regarding time and emotions. The participants' voluntariness and possibility of withdrawing without consequences are emphasized in the information letter, as well as in the interview situation. Regardless of whether the requested person consented, occupational health services were available for those who experienced emotional discomfort. All data are handled in accordance with the General Data Protection Regulation (EU 2016/679) and stored in a secured place that is available only to the research team; the data will be retained for ten years and then disposed of.

The project design has been discussed in research seminars and is preregistered in The Open Science Framework (http://osf.io DOI 10.17605/OSF.IO/Y3P78). Brief feedback regarding the findings will continuously be provided to the included healthcare organizations to enable pressing improvement work. Academic dissemination of the findings will occur through publication in peer-reviewed journals, following the Consolidated criteria for reporting qualitative research (COREQ)[41] or equivalent guidelines. The findings will also be presented at academic and practitioner conferences within the area of quality and safety in healthcare.

DISCUSSION

For peer review only

This multilevel project aims to improve the understanding of how healthcare adapted during the COVID-19 pandemic, the processes involved and the consequences the situations has had on working conditions, ethics and patient safety. A complex adaptive system, such as healthcare, contains many different interacting elements and constantly interacts with the environment.[1 16 17] A complex system's ability to self-organize contributes to its stability and resilience during most disturbances. The pandemic, however, demonstrates how changing dynamics have the potential to challenge and to destabilize the system.[1] Understanding flexibility and adaptive capacities is a main concern for the field of resilience in healthcare.[19 20] Hence, the ongoing pandemic provides unique possibilities to study system-wide resilient performance in real time.

Resilient healthcare is a growing research area,[31 42 43] and theorization has enabled, e.g., the integrated resilience attribute framework,[16] to be developed. However, there is still a need to understand further the interplay between different system levels.[15 16 20 30] Additionally, knowledge about a crisis with the magnitude of the COVID-19 pandemic is lacking. This situation altogether advocates an exploratory research design with openness for the empirical data and main concern of the participants. The current project is thereby underpinned by the methodological assumptions of grounded theory,[32 33 39] which emphasizes theorization built on empiricism. With this method, a flexible yet systematic approach is applied, with openness for what is discovered. The approach enables ideas and concerns that emerge in the early stage of the research to be handled and to be explored further later in the research trajectory. With emphasis on understanding processes, grounded theory[39] provides tools with which to explore complex and continuously changing social contexts and to develop conceptual models or substantive/mid-range theories that can explain the phenomena under study.

For peer review only

When researching the phenomenon of resilience, some core elements should be addressed, namely, the purpose of resilience (resilience for what?), the triggers that activate eventual latent capabilities (resilience to what?), the resources involved (resilience of what?), and the processes supporting resilience (resilience through what?).[21] These core elements align with probing questions used to facilitate analyzing processes in grounded theory, namely, what is going on, when, with whom, and how,[33] in the search for conditions, actions/interactions/reactions, and consequences. The present project explores resilience for the purpose of providing high-quality care. To determine what triggers and activates resilient performance in healthcare during the pandemic, the conditions over time in different contexts and processes that support resilience, adaptive actions, interactions and reactions will be identified and explored. Involved resources (internal and external) will also be identified by exploring different enrolled actors, elements, and affecting factors at different system levels. Finally, as resilience per se is not normative,[20 22] consequences experienced regarding working conditions, ethics and patient safety will be explored as indicators for the ability to provide high-quality care. The described approach may seem sequential; however, the research process is iterative and dynamic.

To our knowledge, this is the first study using grounded theory[33] to study working conditions, ethics and patient safety during a healthcare crisis through a lens of resilience. The approach enables the development of theoretical understanding from empirical data. Additionally, when developing theory, the empirical data will be supplemented with knowledge from the current literature to provide both empirical and theoretical foundations for the development of a substantive theory of resilient performance during the pandemic's rampage. The applied design makes the voice of the people involved in healthcare heard and is anticipated to generate in-depth knowledge that can be used to better understand adaptive capacity and the

For peer review only - http://bmjopen.bmj.com/site/about/guidelines.xhtml

nature and context of adaptations, including the interplay between different system levels, success factors for resilient performance, influencing factors and consequences for sustainable work environment, ethical approach and patient safety. This project can create an important basis for designing interventions with a focus on preparedness to manage current and future challenges in healthcare.

For peer review only

Acknowledgements

The authors would like to acknowledge the healthcare professionals who critically reviewed and gave feedback on the topic guide before the data collection started. We also want to acknowledge all staff and managers in the healthcare organizations that so far contributed to the study by telling their stories and providing the research team with documents to promote our understanding of their situations.

Contributors

PBS was responsible for the study design, the protocol draft and the final version. CG contributed to the study design and drafted substantial parts of the manuscript. MLK, LN and ME provided intellectual input into the study design and contributed to critical review and substantial editing of the draft. ASK, MC, ECM, and MH contributed to critical review and editing of the draft. All authors approved the final version of the manuscript.

Funding

This work was supported by the Centre of Clinical Research Sörmland, Uppsala University, Sweden (Grant No. DLL-940876), and the Regional Research Council in Mid Sweden (Grant No. RFR-939378). Additionally, Region Västmanland, Region Sörmland, Region Dalarna and Dalarna University, Sweden, support the project with in-kind funding.

Competing interests

Non declared.

For peer review only

Ethics approval

The project was approved by the Swedish Ethical Review Authority (Ref. No. 2020-04187).

Patient and public involvement

There was no patient or public involvement in the design and conduct of this study.

Patient consent for publication

Not required.

ORCID iDs

Petronella Bjurling-Sjöberg http://orcid.org/0000-0003-0245-3057

Camilla Göras http://orcid.org/0000-0002-0883-4072

Malin Lohela Karlsson http://orcid.org/0000-0002-9376-849X

Lena Nordgren http://orcid.org/0000-0003-0667-7111

Ann-Sofie Källberg http://orcid.org/0000-0002-0681-9768

Markus Castegren https://orcid.org/0000-0002-9292-0298

Emelie Condén Mellgren http://orcid.org/0000-0003-4525-1623

Mats Holmberg http://orcid.org/0000-0003-1878-0992

Mirjam Ekstedt http://orcid.org/0000-0002-4108-391X

For peer review only

REFERENCES

1. Sturmberg JP, Martin CM. COVID-19 – how a pandemic reveals that everything is connected to everything else. *J Eval Clin Pract* 2020;26(5):1361-67. doi: https://doi.org/10.1111/jep.13419

2. Chua AQ, Tan MMJ, Verma M, et al. Health system resilience in managing the COVID-19 pandemic: lessons from Singapore. *BMJ Global Health* 2020;5(9):e003317. doi: http://doi.org/10.1136/bmjgh-2020-003317

3. Legido-Quigley H, Mateos-García JT, Campos VR, et al. The resilience of the Spanish health system against the COVID-19 pandemic. *Lancet Public Health* 2020;5(5):e251-e52. doi: http://doi.org/10.1016/s2468-2667(20)30060-8

4. Claeson M, Hanson S. COVID-19 and the Swedish enigma. *The Lancet* 2021;397(10271):259-61. https://doi.org/10.1016/S0140-6736(20)32750-1

5. Folkhälsomyndigheten. *Nytt fall av bekräftad COVID-19* [New case of COVID-19, in Swedish]: The Public Health Agency of Sweden. 2020. Available from: https://www.folkhalsomyndigheten.se/nyheter-och-press/nyhetsarkiv/2020/februari/nytt-bekraftat-fall-av-covid-19/

6. Alharbi J, Jackson D, Usher K. The potential for COVID-19 to contribute to compassion fatigue in critical care nurses. *Journal of clinical nursing* 2020;29(15-16):2762-64. doi: http://doi.org/10.1111/jocn.15314

7. Krystal JH. Responding to the hidden pandemic for healthcare workers: stress. *Nature Medicine* 2020;26(5):639. doi: http://doi.org/10.1038/s41591-020-0878-4

8. Vindegaard N, Benros ME. COVID-19 pandemic and mental health consequences: Systematic review of the current evidence. *Brain Behav Immun* 2020;89:531-42. doi: https://doi.org/10.1016/j.bbi.2020.05.048

9. d'Ettorre G, Ceccarelli G, Santinelli L, et al. Post-Traumatic Stress Symptoms in Healthcare Workers Dealing with the COVID-19 Pandemic: A Systematic Review. *International journal of environmental research and public health* 2021;18(2) doi: http://doi.org/10.3390/ijerph18020601

10. Gandhi TK, Singh H. Reducing the Risk of Diagnostic Error in the COVID-19 Era. *Journal of Hospital Medicine* 2020;15(6):363-66. doi: http://doi.org/10.12788/jhm.3461

11. Clarke J, Murray A, Markar SR, et al. New geographic model of care to manage the post-COVID-19 elective surgery aftershock in England: a retrospective observational study. *BMJ Open* 2020;10(10):e042392. doi: http://doi.org/10.1136/bmjopen-2020-042392

12. Stöß C, Steffani M, Kohlhaw K, et al. The COVID-19 pandemic: impact on surgical departments of non-university hospitals. *BMC Surg* 2020;20(1):313. doi: http://doi.org/10.1186/s12893-020-00970-x

13. Sveriges Kommuner och Regioner. *Skador vid vård av covid-19 patienter* [Adverse events in COVID-19 patients, in Swedish]: Swedish Association of Local Authorities and Regions, 2021. Available from: https://webbutik.skr.se/sv/artiklar/skador-vid-vard-av-covid-19-patienter.html

14. Nuzzo JB, Meyer D, Snyder M, et al. What makes health systems resilient against infectious disease outbreaks and natural hazards? Results from a scoping review. *BMC Public Health* 2019;19(1):1310. doi: http://doi.org/10.1186/s12889-019-7707-z

15. Iflaifel M, Lim RH, Ryan K, et al. Resilient Health Care: a systematic review of conceptualisations, study methods and factors that develop resilience. *BMC Health Serv Res* 2020;20(1):324. doi: http://doi.org/10.1186/s12913-020-05208-3

For peer review only

16. Anderson JE, Ross AJ, Macrae C, et al. Defining adaptive capacity in healthcare: A new framework for researching resilient performance. *Appl Ergon* 2020;87:103111. doi: https://doi.org/10.1016/j.apergo.2020.103111

17. Plsek PE, Greenhalgh T. Complexity science: The challenge of complexity in health care. *BMJ Clinical research ed* 2001;323(7313):625-8.

18. Hollnagel E, Braithwaite J, Wears RL. *Resilient health care.* Farnham: Ashgate 2013.

19. Hollnagel E. *Safety-II in practice : developing the resilience potentials.* Abingdon, Oxon: Routledge 2018.

20. Wiig S, Fahlbruch B eds. *Exploring Resilience: A Scientific Journey from Practice to Theory.* Springer Open 2019. Doi: https://doi.org/10.1007/978-3-030-03189-3

21. Wiig S, Aase K, Billett S, et al. Defining the boundaries and operational concepts of resilience in the resilience in healthcare research program. *BMC Health Serv Res* 2020;20(1):330. doi: http://doi.org/10.1186/s12913-020-05224-3

22. Bergström J, Dekker S. Bridging the Macro and the Micro by Considering the Meso: Reflections on the Fractal Nature of Resilience. *Ecology And Society* 2014;19 doi: http://doi.org/10.5751/ES-06956-190422

23. Wears RL, Hollnagel E, Braithwaite J. *Resilient health care Volume 2 The resilience of everyday clinical work*. Farnham, Surrey: Ashgate 2015.

24. Braithwaite J, Churruca, K, Ellis, L.A, Long, J, Clay-Williams, R, Damen, R, Herkes, JPomare, C & Ludlow, K. *Complexity Science in Healthcare - Aspirations, Approaches, Applications and Accomplishments: A white paper*. Australian Institute of Health Innovation, Macquarie University: Sydney, Australia 2017. ISBN: 978-1-74138-456-7

For peer review only

25. Flynn JP, Gascon G, Doyle S, et al. Supporting a Culture of Health in the Workplace: A Review of Evidence-Based Elements. *Am J Health Promot* 2018;32(8):1755-88. doi: http://doi.org/10.1177/0890117118761887

26. Hollnagel E. *Safety-I and safety-II : the past and future of safety management.* Boca Raton: CRC Press 2014.

27. Heath C, Sommerfield A, von Ungern-Sternberg BS. Resilience strategies to manage psychological distress among healthcare workers during the COVID-19 pandemic: a narrative review. *Anaesthesia* 2020;75(10):1364-71. doi: http://doi.org/https://doi.org/10.1111/anae.15180

28. Aase K, Guise V, Billett S, et al. Resilience in Healthcare (RiH): a longitudinal research programme protocol. *BMJ Open* 2020;10(10):e038779. doi: http://doi.org/10.1136/bmjopen-2020-038779

29. Øyri SF, Braut GS, Macrae C, et al. Exploring links between resilience and the macro-level development of healthcare regulation- a Norwegian case study. *BMC Health Serv Res* 2020;20(1):762. doi: http://doi.org/10.1186/s12913-020-05513-x

30. Ellis LA, Churruca K, Clay-Williams R, et al. Patterns of resilience: A scoping review and bibliometric analysis of resilient health care. Safety Science 2019;118:241-57. doi: https://doi.org/10.1016/j.ssci.2019.04.044

31. Berg SH, Akerfjordet K, Ekstedt M, et al. Methodological strategies in resilient health care studies: An integrative review. *Safety Science* 2018;110:300-12. doi: https://doi.org/10.1016/j.ssci.2018.08.025

32. Glaser BG, Strauss AL. *The discovery of grounded theory : strategies for qualitative research.* Chicago: Aldine 1967.

33. Strauss AL, Corbin JM. *Basics of qualitative research : techniques and procedures for developing grounded theory.* Thousand Oaks, Calif.: SAGE 1998.

For peer review only

34. Socialstyrelsen. *About the Swedish healtcare system.* The National Board of Health and Welfare, 2020. Available from https://www.socialstyrelsen.se/en/about-us/healthcare-for-visitors-to-sweden/about-the-swedish-healthcare-system/

35. SPSS Statistics [software]. IBM: New York USA.

36. NVivo [software]. QRS International: Massachusetts USA.

37. Reichertz J. Abduction: The Logic of Discovery of Grounded Theory. *Forum: Qualitative Social Research* 2009;11(1) doi: https://doi.org/10.17169/fqs-11.1.1412

38. Lomborg K, Kirkevold M. Truth and validity in grounded theory -- a reconsidered realist interpretation of the criteria: fit, work, relevance and modifiability. *Nurs Philos* 2003;4(3):189-200. doi: http://doi.org/10.1046/j.1466-769x.2003.00139.x

39. Corbin JM, Strauss AL. *Basics of qualitative research : techniques and procedures for developing grounded theory.* Thousand Oaks: SAGE 2008.

40. Lincoln YS, Guba EG. *Naturalistic inquiry.* Beverly Hills, Calif.: Sage 1985.

41. Tong A, Sainsbury P, Craig J. Consolidated criteria for reporting qualitative research (COREQ): a 32-item checklist for interviews and focus groups. *International Journal for Quality in Health Care* 2007;19(6):349-57. doi: http://doi.org/10.1093/intqhc/mzm042

42. O'Hara JK, Aase K, Waring J. Scaffolding our systems? Patients and families 'reaching in' as a source of healthcare resilience. *BMJ Quality & Safety* 2019;28(1):3-6. doi: http://doi.org/10.1136/bmjqs-2018-008216

43. Smaggus A. Safety-I, Safety-II and burnout: how complexity science can help clinician wellness. *BMJ Quality & Safety* 2019;28(8):667-71. doi: http://doi.org/10.1136/bmjqs-2018-009147

For peer review only

Figure legend

Figure 1 Overview of the project. Illustration of the micro- meso- and macro system levels, time-line and research focus.

For peer review only

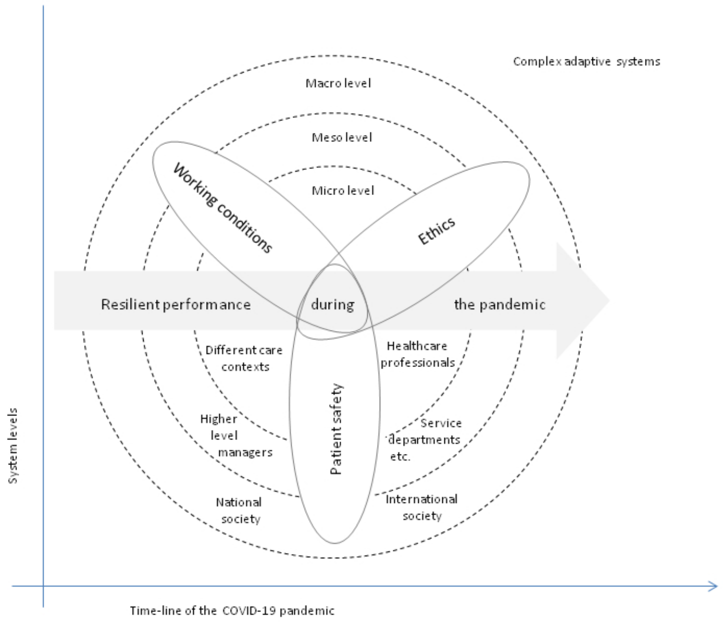

Figure 1 Overview of the project. Illustration of the micro- meso- and macro system levels, time-line and research focus.

254x190mm (96 x 96 DPI)

Standards for Reporting Qualitative Research (SRQR)*

http://www.equator-network.org/reporting-guidelines/srqr/

Title and abstract

	Page/line no(s).
**Title** - Concise description of the nature and topic of the study Identifying the study as qualitative or indicating the approach (e.g., ethnography, grounded theory) or data collection methods (e.g., interview, focus group) is recommended	p2/l6-11
**Abstract** - Summary of key elements of the study using the abstract format of the intended publication; typically includes background, purpose, methods, results, and conclusions	p3-4

Introduction

**Problem formulation** - Description and significance of the problem/phenomenon studied; review of relevant theory and empirical work; problem statement	p6-8
**Purpose or research question** - Purpose of the study and specific objectives or questions	p8/l52 to p9/l27

Methods

**Qualitative approach and research paradigm** - Qualitative approach (e.g., ethnography, grounded theory, case study, phenomenology, narrative research) and guiding theory if appropriate; identifying the research paradigm (e.g., postpositivist, constructivist/ interpretivist) is also recommended; rationale**	p10 + p1[illegible]/l10
**Researcher characteristics and reflexivity** - Researchers' characteristics that may influence the research, including personal attributes, qualifications/experience, relationship with participants, assumptions, and/or presuppositions; potential or actual interaction between researchers' characteristics and the research questions, approach, methods, results, and/or transferability	p17/l10-40
**Context** - Setting/site and salient contextual factors; rationale**	p12/l48 to p13 l29
**Sampling strategy** - How and why research participants, documents, or events were selected; criteria for deciding when no further sampling was necessary (e.g., sampling saturation); rationale**	p13/l36 to p14/l27
**Ethical issues pertaining to human subjects** - Documentation of approval by an appropriate ethics review board and participant consent, or explanation for lack thereof; other confidentiality and data security issues	p17/l47 to p18/l29
**Data collection methods** - Types of data collected; details of data collection procedures including (as appropriate) start and stop dates of data collection and analysis, iterative process, triangulation of sources/methods, and modification of procedures in response to evolving study findings; rationale**	p14/l34 to p15/l54

For peer review only - http://bmjopen.bmj.com/site/about/guidelines.xhtml

**Data collection instruments and technologies** - Description of instruments (e.g., interview guides, questionnaires) and devices (e.g., audio recorders) used for data collection; if/how the instrument(s) changed over the course of the study	p14/l34 to p15 l54
**Units of study** - Number and relevant characteristics of participants, documents, or events included in the study; level of participation (could be reported in results)	n/a
**Data processing** - Methods for processing data prior to and during analysis, including transcription, data entry, data management and security, verification of data integrity, data coding, and anonymization/de-identification of excerpts	p16/l24 to p17/l40
**Data analysis** - Process by which inferences, themes, etc., were identified and developed, including the researchers involved in data analysis; usually references a specific paradigm or approach; rationale**	p16/l24 to p17/l40
**Techniques to enhance trustworthiness** - Techniques to enhance trustworthiness and credibility of data analysis (e.g., member checking, audit trail, triangulation); rationale**	p17/l10-40

Results/findings

**Synthesis and interpretation** - Main findings (e.g., interpretations, inferences, and themes); might include development of a theory or model, or integration with prior research or theory	n/a
**Links to empirical data** - Evidence (e.g., quotes, field notes, text excerpts, photographs) to substantiate analytic findings	n/a

Discussion

**Integration with prior work, implications, transferability, and contribution(s) to the field** - Short summary of main findings; explanation of how findings and conclusions connect to, support, elaborate on, or challenge conclusions of earlier scholarship; discussion of scope of application/generalizability; identification of unique contribution(s) to scholarship in a discipline or field	n/a
**Limitations** - Trustworthiness and limitations of findings	n/a

Other

**Conflicts of interest** - Potential sources of influence or perceived influence on study conduct and conclusions; how these were managed	p22/l24-28
**Funding** - Sources of funding and other support; role of funders in data collection, interpretation, and reporting	p22/l10-20

*The authors created the SRQR by searching the literature to identify guidelines, reporting standards, and critical appraisal criteria for qualitative research; reviewing the reference lists of retrieved sources; and contacting experts to gain feedback. The SRQR aims to improve the transparency of all aspects of qualitative research by providing clear standards for reporting qualitative research.

For peer review only - http://bmjopen.bmj.com/site/about/guidelines.xhtml

**The rationale should briefly discuss the justification for choosing that theory, approach, method, or technique rather than other options available, the assumptions and limitations implicit in those choices, and how those choices influence study conclusions and transferability. As appropriate, the rationale for several items might be discussed together.

Reference:
O'Brien BC, Harris IB, Beckman TJ, Reed DA, Cook DA. **Standards for reporting qualitative research: a synthesis of recommendations.** *Academic Medicine*, Vol. 89, No. 9 / Sept 2014 DOI: 10.1097/ACM.0000000000000388

BMJ Open

Resilient performance in healthcare during the COVID-19 pandemic (ResCOV): study protocol for a multilevel grounded theory study on adaptations, working conditions, ethics and patient safety.

Journal:	*BMJ Open*
Manuscript ID	bmjopen-2021-051928.R1
Article Type:	Protocol
Date Submitted by the Author:	07-Nov-2021
Complete List of Authors:	Bjurling-Sjöberg, Petronella; Uppsala University, Department of Public Health and Caring Sciences, Caring Science; Region Sörmland, Centre for Clinical Research, Uppsala University<br>Göras, Camilla; Region Dalarna, Department of Anesthesia and Intensive Care Unit, Falun Hospital; Dalarna University, School of Health and Welfare<br>Lohela-Karlsson, Malin; Region Västmanland, Centre for Clinical Research, Uppsala University; Uppsala Universitet, Department of Public Health and Caring Sciences<br>Nordgren, Lena; Uppsala University, Department of Public Health and Caring Sciences, Caring Science; Region Sörmland, Centre for Clinical Research Sörmland, Uppsala University<br>Källberg, Ann-Sofie; Dalarna University, School of Health and Welfare; Region Dalarna, Department of Emergency Medicine, Falun Hospital<br>Castegren, Markus; Karolinska Institute, CLINTEC; Region Sörmland, Centre for Clinical Research, Uppsala University<br>Condén Mellgren, Emelie; Region Västmanland, Centre for Clinical Research, Uppsala University<br>Holmberg, Mats; Region Sormland, Centre for Clinical Research Sörmland, Uppsala University; Linneuniversitet, Department of Health and Caring Sciences<br>Ekstedt, Mirjam; Linneuniversitet, Department of Health and Caring Sciences; Karolinska Institutet, LIME
**Primary Subject Heading**:	Health services research
Secondary Subject Heading:	Emergency medicine, Ethics, Intensive care, Nursing, Occupational and environmental medicine
Keywords:	COVID-19, ETHICS (see Medical Ethics), Health & safety < HEALTH SERVICES ADMINISTRATION & MANAGEMENT, QUALITATIVE RESEARCH, Human resource management < HEALTH SERVICES ADMINISTRATION & MANAGEMENT, Quality in health care < HEALTH SERVICES ADMINISTRATION & MANAGEMENT

BMJ

I, the Submitting Author has the right to grant and does grant on behalf of all authors of the Work (as defined in the below author licence), an exclusive licence and/or a non-exclusive licence for contributions from authors who are: i) UK Crown employees; ii) where BMJ has agreed a CC-BY licence shall apply, and/or iii) in accordance with the terms applicable for US Federal Government officers or employees acting as part of their official duties; on a worldwide, perpetual, irrevocable, royalty-free basis to BMJ Publishing Group Ltd ("BMJ") its licensees and where the relevant Journal is co-owned by BMJ to the co-owners of the Journal, to publish the Work in this journal and any other BMJ products and to exploit all rights, as set out in our licence.

The Submitting Author accepts and understands that any supply made under these terms is made by BMJ to the Submitting Author unless you are acting as an employee on behalf of your employer or a postgraduate student of an affiliated institution which is paying any applicable article publishing charge ("APC") for Open Access articles. Where the Submitting Author wishes to make the Work available on an Open Access basis (and intends to pay the relevant APC), the terms of reuse of such Open Access shall be governed by a Creative Commons licence – details of these licences and which Creative Commons licence will apply to this Work are set out in our licence referred to above.

Other than as permitted in any relevant BMJ Author's Self Archiving Policies, I confirm this Work has not been accepted for publication elsewhere, is not being considered for publication elsewhere and does not duplicate material already published. I confirm all authors consent to publication of this Work and authorise the granting of this licence.

For peer review only

TITLE

Resilient performance in healthcare during the COVID-19 pandemic (ResCOV): study protocol for a multilevel grounded theory study on adaptations, working conditions, ethics and patient safety.

Authors

Petronella Bjurling-Sjöberg, CCRN, MSc, PhD [a,b*]

Camilla Göras, RNA, MSc, PhD [c,d]

Malin Lohela Karlsson, PhD [e,a]

Lena Nordgren, RN, MSc, PhD [a,b]

Ann-Sofie Källberg, RN, MSc, PhD [d,f]

Markus Castegren, MD, PhD [g,b]

Emelie Condén Mellgren, RN, PhD [e]

Mats Holmberg, RN, PEN, PhD [b,h]

Mirjam Ekstedt, RN, PhD [h,i]

Author affiliations

[a] Department of Public Health and Caring Sciences, Caring Science, Uppsala University, Uppsala, Sweden

[b] Centre for Clinical Research Sörmland, Uppsala University, Eskilstuna, Sweden

[c] Department of Anesthesia and Intensive Care, Falu Hospital, Region Dalarna, Falun, Sweden

[d] School of Health and Welfare, Dalarna University, Falun, Sweden

[e] Centre for Clinical Research, Region Västmanland, Uppsala University, Västerås, Sweden

[f] Department of Emergency Medicine, Falun Hospital, Falun, Sweden

[g] CLINTEC, Karolinska Institute, Stockholm, Sweden

For peer review only

[h] Department of Health and Caring Sciences, Linnaeus University, Kalmar/Växjö Sweden

[i] Department of Learning, Informatics, Management and Ethics, Karolinska Institute, Stockholm, Sweden.

*Corresponding Author. Postal address: Department of Patient safety, Mälar Hospital, 631 88 Eskilstuna, Sweden; Telephone: +46 76 885 75 92; email address: Petronella.bjurling-sjoberg@pubcare.uu.se

For peer review only

ABSTRACT

Introduction

Since early 2020, the COVID-19 pandemic has challenged societies and revealed the built-in fragility and dependencies in complex adaptive systems, such as healthcare. The pandemic has placed healthcare providers and systems under unprecedented amounts of strain with potential consequences that have not yet been fully elucidated. This multilevel project aims to explore resilient performance with the purpose of improving the understanding of how healthcare has adapted during the pandemic's rampage, the processes involved, and the consequences on working conditions, ethics and patient safety.

Methods

An emerging explorative multilevel design based on grounded theory methodology is applied. Open and theoretical sampling is performed. Empirical data are gathered over time from written narratives and qualitative interviews with staff with different positions in healthcare organizations in two Swedish regions. The participants' first-person stories are complemented with data from the healthcare organizations' internal documents and national and international official documents.

Analysis

Experiences and expressions of resilient performance at different system levels and times, existing influencing risk and success factors at the micro, meso, and macro levels and interrelationships and consequences in different healthcare contexts, are explored using constant comparative analysis. Finally, the-data are complemented with the current literature to develop a substantive theory of resilient performance during the pandemic.

For peer review only

Ethics and dissemination

This project is ethically approved and recognizes the ongoing strain on the healthcare system when gathering data. The ongoing pandemic provides unique possibilities to study system-wide adaptive capacity across different system levels and times, which can create an important basis for designing interventions focusing on preparedness to manage current and future challenges in healthcare. Feedback is provided to the settings to enable pressing improvements. The findings will also be disseminated through scientific journals and conferences.

Registration

The project is preregistered in The Open Science Framework (http://osf.io DOI 10.17605/OSF.IO/PMEXA).

Data Availability Statement

Data (document texts, written narratives and transcripts from interviews without details compromising participants' privacy) will be available from the first author upon reasonable request.

Keywords

Resilience, COVID-19, Grounded theory, Healthcare, Patient safety, Ethics, Worker health, Moral distress

For peer review only

Strengths and limitations of this study

- The first project that uses grounded theory to study working conditions, ethics and patient safety during a healthcare crisis through a lens of resilience.
- Using an exploratory multilevel design with openness for the main concern of the participants, can generate in-depth knowledge to understand better the nature and context of adaptations, the interplay between different system levels, the success factors for resilient performance, and the influencing factors and consequences.
- The project enables the development of theoretical understanding from empirical data, and the findings can create an important basis for designing interventions with a focus on preparedness to manage current and future crises and challenges in healthcare.
- Empirical data are gathered over time from staff from different professions, positions and context in healthcare organizations in two mid-size Swedish regions, which has to be considered when interpreting the findings.
- The first-hand perspective of other stakeholders, such as patients and their families, authorities, financiers and politicians, are not included, which should be further explored in future studies.

For peer review only

INTRODUCTION

Since early 2020, the COVID-19 pandemic has challenged individuals, healthcare systems, and governments, which has revealed the built-in fragility and dependencies in the complex system that constitute current societies.[1] The response to the threat has varied among nations, but overall, the gravity of the situation was initially underestimated.[2-4] In Sweden, the first COVID-19 cases, without connection to travelers, were diagnosed in early March 2020.[5] Calculations regarding progress, including potential effects on healthcare, were soon surpassed as the pandemic hit some regions substantially earlier and harder than predicted, and the need for hospital care increased exponentially. Hence, healthcare organizations rapidly had to escalate the capacity of care to an unprecedented extent. This factor, together with initially limited knowledge about the disease and appropriate treatment, created ad hoc solutions and uncertainty at all levels. In July, the first wave of the pandemic ebbed out, and healthcare could return to relatively ordinary conditions. In autumn, a second wave hit the nation. Additionally, in the spring of 2021, extensive vaccination efforts with unstable vaccine deliveries were organized and manned. Over the past year, the pandemic has forced healthcare organizations and involved staff to be on alert and to frequently adapt to changing conditions. Additionally, at the time of writing, mutations of the virus created a third wave, contributing to further uncertainty about the future.

For peer review only

Although healthcare professionals around the world are used to encountering crises and events, the pandemic has forced them to work under extremely stressful conditions, which has challenged their performance and ability to provide safe care. The severity of the disease and the high in-flow of patients have also meant prioritizing and potentially ethical dilemmas and moral distress. In addition to ethical decisions and facing mortalities far beyond those of previous experience, additional related stressors may include patient overflow, additional

shifts, altered medical interventions, insufficient access to protective equipment and intervening outside the area of expertise.[6 7] Depressive symptoms, anxiety, psychological distress, poor sleep quality[8] and posttraumatic stress disorder[9] have been reported by healthcare professionals. Significant strain on the healthcare system entails an increased risk for diagnostic errors, adverse events and delayed care for patients with COVID-19 as well as other illnesses.[10 11 12] A Swedish medical record review[13] revealed that 18.1% of COVID-19 patients were affected by adverse events during March 2020 to June 2020. This level is twice as high as the frequency for other patients during the same period. Patients admitted to intensive care units were exposed to a four times higher risk of injury.[13] However, experiences and effects of the pandemic are still being elucidated, and knowledge of the processes involved in maintaining safe performance and what support healthcare professionals need in such mega-events is deficient.[14] To generate this additional knowledge, the complexity, interactions and dynamic adaptations that have been made in current societies and healthcare systems need to be considered.[15]

Complex adaptive systems, such as healthcare, are open and constantly interact with their environment. They also contain many elements at different levels that interact in dynamic processes over time.[1 16 17] The micro level of the system, where the patient´s urgent needs require rapid decisions, sometimes operates at different temporalities than the meso- and macro-levels, from which the system's resources and management are controlled and regulated. This issue challenges the ability to anticipate and to understand the larger view of the system and to learn from macro-outcomes in addition to the micro.[18] The ability of a complex adaptive system to self-organize makes it resilient to most disturbances, but changing dynamics also have the potential to destabilize the system.[1] To understand work and

For peer review only

successful responses to challenges and problems in complex adaptive systems, resilience and explicit resilient healthcare have received increased attention.[19 20]

Resilience requires that an organization have the 'potential' for resilient performance, i.e., the capacity to act in specific ways under certain conditions.[19] Resilience in healthcare is defined as 'the capacity to adapt to challenges and changes at different system levels, to maintain high-quality care'.[21] This perspective involves a proactive approach, which requires the potential to anticipate, to monitor, to learn and to respond.[19] However, resilience is not normative, as adaptations also have the potential for negative consequences.[20] Safety science warns that reducing resilient performance to individuals' capacity to face challenges and complexity may lead to the creation of safety strategies that rely primarily on individuals, and not on system safety.[22 23]

Today, patient safety is often assessed through analysis and the quantification of deviations. By accepting that healthcare is a complex adaptive system and adopting a system perspective with a focus on preventing and strengthening success, a more proactive approach emerges.[24] Such an approach may improve patient safety and quality of care, prevent worker ill health and achieve good working conditions. However, although there has been an increased focus on proactive safety work, success factors regarding working conditions and patient safety are still relatively unexplored.[24 25] Additionally, despite the importance of learning, success factors for adaptive capacity are relatively unexplored,[26] and knowledge about how resilient performance is created is still deficient.[14 15 24 27] Except for some notable ongoing studies,[28 29] empirical studies on resilience have focused mainly on the micro level.[30 31] To extend the understanding of resilient performance and to explain important interactions between different

For peer review only

system levels in healthcare, there is a need to contribute to the theory of resilient healthcare with empirical data from a multilevel perspective.[20 31]

The pandemic has placed healthcare providers and systems under unprecedented strain, with potential consequences on working conditions, ethics and patient safety that have not yet been fully elucidated. The adaptive capacity of a complex adaptive system under extreme pressure can never be fully understood. However, the ongoing pandemic provides unique possibilities to study system-wide adaptive capacity in real time. Applying a multilevel approach and studying how professionals at different levels of the system adapt their operations under the ongoing pandemic will increase the understanding of how adaptive processes interplay across care contexts and system levels to sustain high-quality performance and safety under extraordinary conditions. This approach will also increase knowledge about useful adaptations and success factors for how resilience is created through rapid decisions and adaptations at the micro level of the system and in the interplay between the micro- and meso-level. The staff´s experiences of ethical stress and workload will also be explored. Thus, empirical exploration of resilient performance, influencing factors, and consequences across different system levels and times can create an important basis for designing interventions focusing on preparedness to manage current and future challenges in healthcare.

Aims and objectives

This study protocol describes the research project ‘Resilient performance in healthcare during the COVID-19 pandemic (ResCOV)’. The overall aim of this multilevel project is to improve the understanding of how healthcare adapted during the pandemic’s rampage, the processes

For peer review only

involved, and the consequences on working conditions, ethics and patient safety. The specific objectives and research questions that guide the project are as follows:

I. To explore and describe how staff from different professions and positions in healthcare organizations experienced and managed the pandemic's rampage and thus influenced factors and consequences.

   i. What were the main challenges for staff at different levels in the healthcare organizations during the initial phase of the pandemic?

   ii. How were the challenges addressed and coped with in different types of care contexts (i.e. intensive care, ambulance care, emergency care, and specific COVID units)?

   iii. How did the main challenges and responses change over time during the continuing rampage of the pandemic?

   iv. What conditions (internal and external) influenced the responses in different care contexts over time, and with what consequences?

II. To explore and identify expressions of resilient performance at different system levels and times, influencing risk and success factors at the micro-meso-macro levels, the interrelationships and consequences on sustainable working conditions, and ethics and patient safety in different healthcare contexts.

   i. How did capacity to monitor, anticipate-and adapt in response to changing work demands, ethical and patient safety challenges develop over time in different contexts at micro level and at meso level?

   ii. What risk and success factors on different levels influenced the micro- and meso-levels response?

For peer review only

iii. What lessons were learned, and what potential improvements were implemented?

iv. How did adaptive processes interplay across care contexts and system levels (micro-meso-macro), and with what consequences?

III. To develop a substantive theory to provide a comprehensive contextualized explanation of resilient performance within healthcare during the pandemic.

i. What were the overall triggers that activated capabilities to respond?

ii. What were the overall responses in terms of adaptive actions, interactions and reactions to monitor, anticipate, prepare and learn over time?

iii. What conditions and processes supported or undermined successful responses, and how did different system levels interplay?

iv. What was the outcome in terms of consequences on working conditions, ethics and patient safety?

METHODS AND ANALYSIS

Overall design

To explore resilient performance during the COVID-19 pandemic's rampage, this project has an emerging explorative multilevel design based on the grounded theory methodology[32 33] (see Figure 1). This type of design is ideal when the aim is to understand phenomena in a research area where previous knowledge is limited and where the concerns of people involved are in focus. The project started in September 2020. To cover the first, second and third wave of COVID-19, including the aftermath, data gathering from staff in the included healthcare organizations is estimated to continue until December 2022. The end of the project is however

For peer review only

depending on when theoretical saturation is achieved, i.e., when sufficiently comprehensive data to thoroughly understand the characteristics of the phenomenon and process is obtained.[32 33] To address the overall aim and the three study objectives, the research will be conducted in four iterative, flexible and closely integrated cycles of activities, including staff from different professions and levels in healthcare organizations, complemented with data from different documents, as specified below.

Cycle I, micro level: To explore how healthcare professionals experienced and managed the pandemic's rampage, empirical data from professionals working with COVID-19 patients in different contexts, such as emergency departments, specific COVID units and intensive care, will be gathered. The datasets will be analyzed separately and focus on each profession at the time. Interactions with nurse managers and medical directors, as well as interprofessional interactions, influencing factors and consequences in different phases of the pandemic, will also be analyzed in the first cycle.

Cycle II, meso level: Empirical data from the staff in positions not directly involved in patient care, such as managers at the meso level and service departments etc., will be gathered and analyzed; the focus will be on their experiences, i.e., interorganizational interactions, decisions and adaptations, influencing factors and the consequences on micro- and meso-level performance at different phases of the pandemic.

Cycle III, multilevel (micro-macro): Empirical data from key informants in context indirectly affected by the pandemic, temporary disaster management of healthcare organizations, internal documents from healthcare organizations, and official national and international documents will be gathered and integrated with the findings from cycles I and II. These integrated data will be analyzed cross-sectionally and consider the different system levels and phases of the pandemic by focusing on resilient performance, the influencing

For peer review only - http://bmjopen.bmj.com/site/about/guidelines.xhtml

factors and the consequences in each of the areas of specific interest, namely, sustainable working conditions, ethics and patient safety.

Cycle IV, multilevel (micro-macro): Finally, we will develop a comprehensive contextualized explanation of resilient performance within healthcare during the pandemic. The findings from cycles I-III will be synthesized and supplemented with knowledge from the current literature to provide an empirical and theoretical foundation for the development of a substantive theory of resilient performance during the pandemic rampage.

Please, insert Fig. 1

Study context

Swedish public healthcare provide the setting for the project. Healthcare in Sweden is largely publicly funded. National regulations provide a foundation for the healthcare system, but the management is self-governed by the 21 regional councils. Therefore, each region is responsible for the organization and prioritization of healthcare resources.[34] The clinical healthcare professionals mainly include registered nurses, some who are specialized within certain fields, e.g., intensive care and anesthesiology; undergraduate assistant nurses; physicians specialized within certain fields, such as medicine, surgery or anesthesiology, and those under training; and some allied healthcare professions, such as physiotherapists, occupational therapists, dieticians and counselors. Unlike some other countries, drugs and mechanical ventilation are handled within the healthcare team without pharmacists or respiratory therapists.

For peer review only

The project is performed in the healthcare organizations of two Swedish regions, including seven somatic hospitals and outpatient care. The regions each have a population of ~300 000, which is approximately the median size for the nation. The total number of in-hospital beds in each region is 500-600, with 70-300 beds/hospital, which is a common size of local or county hospitals in the nation.

Sample and Participants

Open sampling and theoretical sampling[33] are performed with the main data source being staff from the included healthcare organizations (micro- and meso-levels). This empirical data is complemented with data from applicable documents (micro-meso-macro levels), as specified below. Recruitment started September 2020 and is ongoing. Potential participants are contacted through the organizations' e-mail systems. They are informed about the study, aim, procedure and voluntariness and asked about participation.

Initially, aiming to maximize variations in experiences in cycles I-III, open sampling[33] was conducted by recruiting staff from different professions, positions and contexts in healthcare organizations. First, healthcare professionals (registered nurses, assistant nurses, physicians, allied healthcare professions and clinical managers) were recruited who, on a regular or temporary basis, were on duty in the sectors of healthcare that were most affected by the first wave of the pandemic, including emergency departments, specific COVID units, intensive care and operating room departments. Second, higher-level managers were recruited from all parts of the hospitals and primary care, key informants from selected service and support departments, and top-level management in the healthcare organizations and temporary

For peer review only

disaster management. At the time of this writing, March 2021, just over 200 participants have been included.

Later, guided by the simultaneously performed analysis in cycle I-IV, theoretical sampling[33] take place, and data are gathered to explore differences over time, to fill gaps in understanding and to saturate emerging concepts and categories. In this phase, we return to already recruited participants for complementary information and recruit additional participants from the healthcare organizations if needed. Additionally, data are utilized from applicable internal documents from the included healthcare organizations (micro- and meso-levels), such as statistics, guidelines, protocols, and applicable official national and international documents (macro level), such as statistics, information, guidelines, recommendations, and legal regulations. The inclusion of data from these documents aims to provide enhanced understanding of different factors at the micro-meso-macro levels that influenced the conditions in the included healthcare organizations and thereby had potential consequences on working conditions, ethics and patient safety. Thereby inclusion of documents is guided by the analysis of the participants' first-person experiences.

Data collection

Primary data consist of the participants´ first-person written or verbal narratives, which are supplemented with data from documents as specified above. Data collection started simultaneously with recruitment (September 2020) and will continue until theoretical saturation[32 33] is achieved in all parts of the project. Individuals in the included healthcare organizations who consent to participate are given the choice to tell their story in writing or to participate in individual qualitative interviews. They are also asked to fill out a questionnaire

For peer review only

for some demographic data (gender, age, occupation and years in this occupation), regular workplace, role and duties, and workplace, role and duties during the pandemic. Additionally, participants are asked to attach supplementary documents from their workplace that they think could facilitate the researchers understanding of their story and to provide contact information if they consent to be contacted again for further questions.

The participants are encouraged to tell their story about their experiences, thoughts and feelings during the pandemic openly. To aid the written narrative, a study-specific guide has been created based on a review of publications and the researchers' experiences. The face validity of the guide was tested by six healthcare professionals, and after minor revisions it was assessed as valid. The guide includes some guiding questions for the topics of specific interest (working conditions, ethics, patient safety, adaptations, influencing factors, consequences and lessons learned) but urges the participants to tell or to leave out whatever they want.

The interviews are performed by the researchers with great openness for what the informant thinks is important to illuminate, starting with the question 'Can you please tell me how the situation been during the pandemic?' and continuing with illuminating probing questions. A semistructured interview guide has been developed, which includes the same topics as those found in the guide for the written narratives; however, this guide is used in a flexible manner to ensure that the participants' concerns are captured. Based on the ongoing analysis, both guides are successively refined to support exploration of emerging understandings. All interviews are digitally recorded and transcribed verbatim.

For peer review only

Data collection from documents on different system levels and periods of time, as described above, are conducted first through the participants who provide internal documents from their workplace (micro- and meso-levels). Second, based on needs that emerge in the analysis, the researchers request useful official documents from the included healthcare organizations (micro- and meso-levels) and applicable authorities (macro level) or collect them from their websites if available.

Data management and analysis

Demographic data are managed in SPSS Statistics[35] to provide descriptive statistics of the participants. All qualitative data are managed in NVivo[36] and analyzed using constant comparative analysis according to the principles of grounded theory.[33] The research questions guide the analysis but are open and modifiable to what emerge in data as significant. The analysis process started with the first data collection and will continue until theoretical saturation is achieved in all parts of the project.

Throughout the analysis process, data and emerging concepts and categories are compared back and forth, moving between open, axial and selective coding as new data are collected and the theorizing proceeds. The theorizing includes reflecting, writing memos and drawing diagrams of interpretations, ideas, assumed associations and theoretical reflections related to each emerging category, moving towards explanation in an abductive approach,[37] shifting between inductive interpretation and deductive testing as hypotheses evolve. Tools, such as the ‘paradigm model’ to facilitate analysis of process and the ‘contextual matrix’ to facilitate analysis of the different system levels,[33] are utilized in a flexible manner to facilitate accuracy

For peer review only

without restricting the creativity of the analysis. Finally, the developed theory are validated in the original empirical raw data.

In cycles I and II, data are analyzed as described above in the different studies, focusing on different contexts and levels of the included healthcare organizations, with different professions, interactions, adaptations, influencing factors, and consequences, in each of the specific areas of interest (i.e. sustainable working conditions, ethics, and patient safety) and phases of the pandemic and aftermath. Findings from cycle I and II (micro- and meso-levels) are then, in cycle III, complemented with additional data from participants and documents (meso- and macro-levels), and analyzed cross-sectionally, considering the different system levels and phases of the pandemic, to develop further the understanding of the complexity, including interactions and dynamic adoptions that been made in the included healthcare organizations, influencing factors within the organizations, influencing factors from the macro level, and the consequences on working conditions, ethics, and patient safety. Finally, in cycle IV, when the pandemic and aftermath have been completed, the findings from all the previous cycles are supplemented with knowledge from the current literature to provide an empirical and theoretical foundation for developing a substantive theory of resilient performance during the pandemic rampage. Hence, the cycles of the project are closely integrated and, although not linear, built upon each other and enable theory to evolve.

The development of theoretical understanding is a process between the researcher and the empirical data, and the voice of the participants is rendered into the findings. The level of abstraction/theorization in the studies depends on the content and depth of the data obtained. Hence, the research team is open to settling with a lower level of abstraction, such as purely

For peer review only

descriptive results or conceptual modeling in some studies. These results can also be of substantial usefulness. The most important quality criterion in grounded theory is that the findings are truly grounded in the data. The findings should 'fit' the empirical situations in the social context under study, 'work' to explain what was going on in the studied context and have 'relevance' by representing subjects of real concern to the people involved. Finally, the developed theory or conceptual model should have 'modifiability', meaning that as changes take place in reality and studies are performed in other contexts, the theory can evolve.[33 38]

Based on the philosophical orientation of the project, reality is multiple, complex, socially constructed, subjectively perceived, and can be interpreted but not fully known. A further assumption is that knowledge is created within the interaction between the researchers and the participants. Consequently, objectivity is not desirable. However, to ensure that the findings are grounded in the empirical data, the researchers have to be reflective regarding their own pre-understanding.[39] Strategies appropriate for qualitative studies are applied to achieve trustworthiness.[40] The primary analysis in each study will be conducted by two researchers, and reflective discussions will take place with other members of the research team. Clinical experiences within the research team enable theoretical sensibility. Credibility is enhanced through an audit trail, collaboration in the analysis, comprehensive memo writing, and reflective discussions within the research group, which include individuals without experience of care during the pandemic, an audit trail, peer reviews and in-depth methodological descriptions. The transferability of the findings will be enhanced by in-depth contextual descriptions.

Patient and public involvement

There was no patient or public involvement in the design and conduct of this study.

For peer review only

ETHICS AND DISSEMINATION

The project was approved by the Swedish Ethical Review Authority (Ref. No. 2020-04187). The continuing effect of the pandemic on healthcare means that the researchers have to be responsive to the participants' conditions, both regarding time and emotions. The participants' voluntariness and possibility of withdrawing without consequences are emphasized in the information letter, as well as in the interview situation. Regardless of whether the requested person consented, occupational health services were available for those who experienced emotional discomfort. All data are handled in accordance with the General Data Protection Regulation (EU 2016/679) and stored in a secured place that is available only to the research team; the data will be retained for ten years and then disposed of.

The project design has been discussed in research seminars and is preregistered in The Open Science Framework (http://osf.io DOI 10.17605/OSF.IO/PMEXA). Brief feedback regarding the findings will continuously be provided to the included healthcare organizations to enable pressing improvement work. Academic dissemination of the findings will occur through publication in peer-reviewed journals, following the Consolidated criteria for reporting qualitative research (COREQ)[41] or equivalent guidelines. The findings will also be presented at academic and practitioner conferences within the area of quality and safety in healthcare.

DISCUSSION

For peer review only

This multilevel project aims to improve the understanding of how healthcare adapted during the COVID-19 pandemic, the processes involved and the consequences the situations has had on working conditions, ethics and patient safety. A complex adaptive system, such as healthcare, contains many different interacting elements and constantly interacts with the environment.[1 16 17] A complex system's ability to self-organize contributes to its stability and resilience during most disturbances. The pandemic, however, demonstrates how changing dynamics have the potential to challenge and to destabilize the system.[1] Understanding flexibility and adaptive capacities is a main concern for the field of resilience in healthcare.[19 20] Hence, the ongoing pandemic provides unique possibilities to study system-wide resilient performance in real time.

Resilient healthcare is a growing research area,[31 42 43] and theorization has enabled, e.g., the integrated resilience attribute framework,[16] to be developed. However, there is still a need to understand further the interplay between different system levels.[15 16 20 30] Additionally, knowledge about a crisis with the magnitude of the COVID-19 pandemic is lacking. This situation altogether advocates an exploratory research design with openness for the empirical data and main concern of the participants. The current project is thereby underpinned by the methodological assumptions of grounded theory,[32 33 39] which emphasizes theorization built on empiricism. With this method, a flexible yet systematic approach is applied, with openness for what is discovered. The approach enables ideas and concerns that emerge in the early stage of the research to be handled and to be explored further later in the research trajectory. With emphasis on understanding processes, grounded theory[39] provides tools with which to explore complex and continuously changing social contexts and to develop conceptual models or substantive/mid-range theories that can explain the phenomena under study.

For peer review only

When researching the phenomenon of resilience, some core elements should be addressed, namely, the purpose of resilience (resilience for what?), the triggers that activate eventual latent capabilities (resilience to what?), the resources involved (resilience of what?), and the processes supporting resilience (resilience through what?).[21] These core elements align with probing questions used to facilitate analyzing processes in grounded theory, namely, what is going on, when, with whom, and how,[33] in the search for conditions, actions/interactions/reactions, and consequences. The present project explores resilience for the purpose of providing high-quality care. To determine what triggers and activates resilient performance in healthcare during the pandemic, the conditions over time in different contexts and processes that support resilience, adaptive actions, interactions and reactions will be identified and explored. Involved resources (internal and external) will also be identified by exploring different enrolled actors, elements, and affecting factors at different system levels. Finally, as resilience per se is not normative,[20 22] consequences experienced regarding working conditions, ethics and patient safety will be explored as indicators for the ability to provide high-quality care. The described approach may seem sequential; however, the research process is iterative and dynamic.

The project has limitations that should be acknowledged. Firstly, the studies are performed in the healthcare organizations in two Swedish regions only. However, these regions were heavily affected by the pandemic, and transferability of the findings will be enhanced by in-depth contextual descriptions. Secondly, although complemented with internal documents from the healthcare organizations, and official national and international documents, data are mainly gathered from staff. The first-hand perspective of other stakeholders, such as patients and their families, authorities, financiers and politicians, are not included. Further studies should explore the phenomenon in other settings and also preferably include other

For peer review only

stakeholders. Thirdly, the project is inevitably affected by the progress of the COVID-19 pandemic and the staff's ability to participate; therefore, the research trajectory is difficult to predict precisely.

To our knowledge, this is the first study using grounded theory[33] to study working conditions, ethics and patient safety during a healthcare crisis through a lens of resilience. The approach enables the development of theoretical understanding from empirical data. Additionally, when developing theory, the empirical data will be supplemented with knowledge from the current literature to provide both empirical and theoretical foundations for the development of a substantive theory of resilient performance during the pandemic's rampage. The applied design makes the voice of the people involved in healthcare heard and is anticipated to generate in-depth knowledge that can be used to better understand adaptive capacity and the nature and context of adaptations, including the interplay between different system levels, success factors for resilient performance, influencing factors and consequences for sustainable work environment, ethical approach and patient safety. This project can create an important basis for designing interventions with a focus on preparedness to manage current and future challenges in healthcare.

For peer review only

Acknowledgements

The authors would like to acknowledge the healthcare professionals who critically reviewed and gave feedback on the topic guide before the data collection started. We also want to acknowledge all staff and managers in the healthcare organizations that so far contributed to the study by telling their stories and providing the research team with documents to promote our understanding of their situations.

Contributors

PBS was responsible for the study design, the protocol draft and the final version. CG contributed to the study design and drafted substantial parts of the manuscript. MLK, LN and ME provided intellectual input into the study design and contributed to critical review and substantial editing of the draft. ASK, MC, ECM, and MH contributed to critical review and editing of the draft. All authors approved the final version of the manuscript.

Funding

This work was supported by the Centre of Clinical Research Sörmland, Uppsala University, Sweden (Grant No. DLL-940876), and the Regional Research Council in Mid Sweden (Grant No. RFR-939378). Additionally, Region Västmanland, Region Sörmland, Region Dalarna and Dalarna University, Sweden, support the project with in-kind funding.

Competing interests

Non declared.

Ethics approval

For peer review only

The project was approved by the Swedish Ethical Review Authority (Ref. No. 2020-04187).

Patient consent for publication

Not required.

ORCID iDs

Petronella Bjurling-Sjöberg http://orcid.org/0000-0003-0245-3057

Camilla Göras http://orcid.org/0000-0002-0883-4072

Malin Lohela Karlsson http://orcid.org/0000-0002-9376-849X

Lena Nordgren http://orcid.org/0000-0003-0667-7111

Ann-Sofie Källberg http://orcid.org/0000-0002-0681-9768

Markus Castegren https://orcid.org/0000-0002-9292-0298

Emelie Condén Mellgren http://orcid.org/0000-0003-4525-1623

Mats Holmberg http://orcid.org/0000-0003-1878-0992

Mirjam Ekstedt http://orcid.org/0000-0002-4108-391X

For peer review only

REFERENCES

1. Sturmberg JP, Martin CM. COVID-19 – how a pandemic reveals that everything is connected to everything else. *J Eval Clin Pract* 2020;26(5):1361-67. doi: https://doi.org/10.1111/jep.13419

2. Chua AQ, Tan MMJ, Verma M, et al. Health system resilience in managing the COVID-19 pandemic: lessons from Singapore. *BMJ Global Health* 2020;5(9):e003317. doi: http://doi.org/10.1136/bmjgh-2020-003317

3. Legido-Quigley H, Mateos-García JT, Campos VR, et al. The resilience of the Spanish health system against the COVID-19 pandemic. *Lancet Public Health* 2020;5(5):e251-e52. doi: http://doi.org/10.1016/s2468-2667(20)30060-8

4. Claeson M, Hanson S. COVID-19 and the Swedish enigma. *The Lancet* 2021;397(10271):259-61. https://doi.org/10.1016/S0140-6736(20)32750-1

5. Folkhälsomyndigheten. *Nytt fall av bekräftad COVID-19* [New case of COVID-19, in Swedish]: The Public Health Agency of Sweden. 2020. Available from: https://www.folkhalsomyndigheten.se/nyheter-och-press/nyhetsarkiv/2020/februari/nytt-bekraftat-fall-av-covid-19/

6. Alharbi J, Jackson D, Usher K. The potential for COVID-19 to contribute to compassion fatigue in critical care nurses. *Journal of clinical nursing* 2020;29(15-16):2762-64. doi: http://doi.org/10.1111/jocn.15314

7. Krystal JH. Responding to the hidden pandemic for healthcare workers: stress. *Nature Medicine* 2020;26(5):639. doi: http://doi.org/10.1038/s41591-020-0878-4

8. Vindegaard N, Benros ME. COVID-19 pandemic and mental health consequences: Systematic review of the current evidence. *Brain Behav Immun* 2020;89:531-42. doi: https://doi.org/10.1016/j.bbi.2020.05.048

For peer review only

9. d'Ettorre G, Ceccarelli G, Santinelli L, et al. Post-Traumatic Stress Symptoms in Healthcare Workers Dealing with the COVID-19 Pandemic: A Systematic Review. *International journal of environmental research and public health* 2021;18(2) doi: http://doi.org/10.3390/ijerph18020601

10. Gandhi TK, Singh H. Reducing the Risk of Diagnostic Error in the COVID-19 Era. *Journal of Hospital Medicine* 2020;15(6):363-66. doi: http://doi.org/10.12788/jhm.3461

11. Clarke J, Murray A, Markar SR, et al. New geographic model of care to manage the post-COVID-19 elective surgery aftershock in England: a retrospective observational study. *BMJ Open* 2020;10(10):e042392. doi: http://doi.org/10.1136/bmjopen-2020-042392

12. Stöß C, Steffani M, Kohlhaw K, et al. The COVID-19 pandemic: impact on surgical departments of non-university hospitals. *BMC Surg* 2020;20(1):313. doi: http://doi.org/10.1186/s12893-020-00970-x

13. Sveriges Kommuner och Regioner. *Skador vid vård av covid-19 patienter* [Adverse events in COVID-19 patients, in Swedish]: Swedish Association of Local Authorities and Regions, 2021. Available from: https://webbutik.skr.se/sv/artiklar/skador-vid-vard-av-covid-19-patienter.html

14. Nuzzo JB, Meyer D, Snyder M, et al. What makes health systems resilient against infectious disease outbreaks and natural hazards? Results from a scoping review. *BMC Public Health* 2019;19(1):1310. doi: http://doi.org/10.1186/s12889-019-7707-z

15. Iflaifel M, Lim RH, Ryan K, et al. Resilient Health Care: a systematic review of conceptualisations, study methods and factors that develop resilience. *BMC Health Serv Res* 2020;20(1):324. doi: http://doi.org/10.1186/s12913-020-05208-3

For peer review only

16. Anderson JE, Ross AJ, Macrae C, et al. Defining adaptive capacity in healthcare: A new framework for researching resilient performance. *Appl Ergon* 2020;87:103111. doi: https://doi.org/10.1016/j.apergo.2020.103111

17. Plsek PE, Greenhalgh T. Complexity science: The challenge of complexity in health care. *BMJ Clinical research ed* 2001;323(7313):625-8.

18. Hollnagel E, Braithwaite J, Wears RL. *Resilient health care.* Farnham: Ashgate 2013.

19. Hollnagel E. *Safety-II in practice : developing the resilience potentials.* Abingdon, Oxon: Routledge 2018.

20. Wiig S, Fahlbruch B eds. *Exploring Resilience: A Scientific Journey from Practice to Theory.* Springer Open 2019. Doi: https://doi.org/10.1007/978-3-030-03189-3

21. Wiig S, Aase K, Billett S, et al. Defining the boundaries and operational concepts of resilience in the resilience in healthcare research program. *BMC Health Serv Res* 2020;20(1):330. doi: http://doi.org/10.1186/s12913-020-05224-3

22. Bergström J, Dekker S. Bridging the Macro and the Micro by Considering the Meso: Reflections on the Fractal Nature of Resilience. *Ecology And Society* 2014;19 doi: http://doi.org/10.5751/ES-06956-190422

23. Wears RL, Hollnagel E, Braithwaite J. *Resilient health care Volume 2 The resilience of everyday clinical work*. Farnham, Surrey: Ashgate 2015.

24. Braithwaite J, Churruca, K, Ellis, L.A, Long, J, Clay-Williams, R, Damen, R, Herkes, JPomare, C & Ludlow, K. *Complexity Science in Healthcare - Aspirations, Approaches, Applications and Accomplishments: A white paper*. Australian Institute of Health Innovation, Macquarie University: Sydney, Australia 2017. ISBN: 978-1-74138-456-7

For peer review only

25. Flynn JP, Gascon G, Doyle S, et al. Supporting a Culture of Health in the Workplace: A Review of Evidence-Based Elements. *Am J Health Promot* 2018;32(8):1755-88. doi: http://doi.org/10.1177/0890117118761887

26. Hollnagel E. *Safety-I and safety-II : the past and future of safety management.* Boca Raton: CRC Press 2014.

27. Heath C, Sommerfield A, von Ungern-Sternberg BS. Resilience strategies to manage psychological distress among healthcare workers during the COVID-19 pandemic: a narrative review. *Anaesthesia* 2020;75(10):1364-71. doi: http://doi.org/https://doi.org/10.1111/anae.15180

28. Aase K, Guise V, Billett S, et al. Resilience in Healthcare (RiH): a longitudinal research programme protocol. *BMJ Open* 2020;10(10):e038779. doi: http://doi.org/10.1136/bmjopen-2020-038779

29. Øyri SF, Braut GS, Macrae C, et al. Exploring links between resilience and the macro-level development of healthcare regulation- a Norwegian case study. *BMC Health Serv Res* 2020;20(1):762. doi: http://doi.org/10.1186/s12913-020-05513-x

30. Ellis LA, Churruca K, Clay-Williams R, et al. Patterns of resilience: A scoping review and bibliometric analysis of resilient health care. Safety Science 2019;118:241-57. doi: https://doi.org/10.1016/j.ssci.2019.04.044

31. Berg SH, Akerfjordet K, Ekstedt M, et al. Methodological strategies in resilient health care studies: An integrative review. *Safety Science* 2018;110:300-12. doi: https://doi.org/10.1016/j.ssci.2018.08.025

32. Glaser BG, Strauss AL. *The discovery of grounded theory : strategies for qualitative research.* Chicago: Aldine 1967.

33. Strauss AL, Corbin JM. *Basics of qualitative research : techniques and procedures for developing grounded theory.* Thousand Oaks, Calif.: SAGE 1998.

For peer review only

34. Socialstyrelsen. *About the Swedish healtcare system.* The National Board of Health and Welfare, 2020. Available from https://www.socialstyrelsen.se/en/about-us/healthcare-for-visitors-to-sweden/about-the-swedish-healthcare-system/

35. SPSS Statistics [software]. IBM: New York USA.

36. NVivo [software]. QRS International: Massachusetts USA.

37. Reichertz J. Abduction: The Logic of Discovery of Grounded Theory. *Forum: Qualitative Social Research* 2009;11(1) doi: https://doi.org/10.17169/fqs-11.1.1412

38. Lomborg K, Kirkevold M. Truth and validity in grounded theory -- a reconsidered realist interpretation of the criteria: fit, work, relevance and modifiability. *Nurs Philos* 2003;4(3):189-200. doi: http://doi.org/10.1046/j.1466-769x.2003.00139.x

39. Corbin JM, Strauss AL. *Basics of qualitative research : techniques and procedures for developing grounded theory.* Thousand Oaks: SAGE 2008.

40. Lincoln YS, Guba EG. *Naturalistic inquiry.* Beverly Hills, Calif.: Sage 1985.

41. Tong A, Sainsbury P, Craig J. Consolidated criteria for reporting qualitative research (COREQ): a 32-item checklist for interviews and focus groups. *International Journal for Quality in Health Care* 2007;19(6):349-57. doi: http://doi.org/10.1093/intqhc/mzm042

42. O'Hara JK, Aase K, Waring J. Scaffolding our systems? Patients and families 'reaching in' as a source of healthcare resilience. *BMJ Quality & Safety* 2019;28(1):3-6. doi: http://doi.org/10.1136/bmjqs-2018-008216

43. Smaggus A. Safety-I, Safety-II and burnout: how complexity science can help clinician wellness. *BMJ Quality & Safety* 2019;28(8):667-71. doi: http://doi.org/10.1136/bmjqs-2018-009147

For peer review only

Figure legend

Figure 1 Overview of the project. Illustration of the micro- meso- and macro system levels, time-line and research focus.

For peer review only

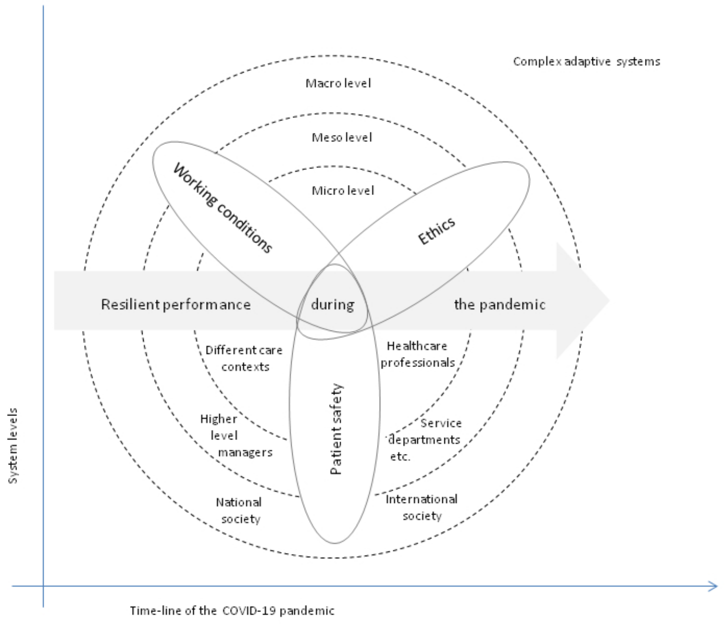

Figure 1 Overview of the project. Illustration of the micro- meso- and macro system levels, time-line and research focus.

254x190mm (96 x 96 DPI)

Standards for Reporting Qualitative Research (SRQR)*

http://www.equator-network.org/reporting-guidelines/srqr/

Title and abstract

	Page/line no(s).
**Title** - Concise description of the nature and topic of the study Identifying the study as qualitative or indicating the approach (e.g., ethnography, grounded theory) or data collection methods (e.g., interview, focus group) is recommended	p2/l6-11
**Abstract** - Summary of key elements of the study using the abstract format of the intended publication; typically includes background, purpose, methods, results, and conclusions	p3-4

Introduction

**Problem formulation** - Description and significance of the problem/phenomenon studied; review of relevant theory and empirical work; problem statement	p6-8
**Purpose or research question** - Purpose of the study and specific objectives or questions	p8/l52 to p9/l27

Methods

**Qualitative approach and research paradigm** - Qualitative approach (e.g., ethnography, grounded theory, case study, phenomenology, narrative research) and guiding theory if appropriate; identifying the research paradigm (e.g., postpositivist, constructivist/ interpretivist) is also recommended; rationale**	p10 + p17/l10
**Researcher characteristics and reflexivity** - Researchers' characteristics that may influence the research, including personal attributes, qualifications/experience, relationship with participants, assumptions, and/or presuppositions; potential or actual interaction between researchers' characteristics and the research questions, approach, methods, results, and/or transferability	p17/l10-40
**Context** - Setting/site and salient contextual factors; rationale**	p12/l48 to p13 l29
**Sampling strategy** - How and why research participants, documents, or events were selected; criteria for deciding when no further sampling was necessary (e.g., sampling saturation); rationale**	p13/l36 to p14/l27
**Ethical issues pertaining to human subjects** - Documentation of approval by an appropriate ethics review board and participant consent, or explanation for lack thereof; other confidentiality and data security issues	p17/l47 to p18/l29
**Data collection methods** - Types of data collected; details of data collection procedures including (as appropriate) start and stop dates of data collection and analysis, iterative process, triangulation of sources/methods, and modification of procedures in response to evolving study findings; rationale**	p14/l34 to p15/l54

For peer review only - http://bmjopen.bmj.com/site/about/guidelines.xhtml

**Data collection instruments and technologies** - Description of instruments (e.g., interview guides, questionnaires) and devices (e.g., audio recorders) used for data collection; if/how the instrument(s) changed over the course of the study	p14/l34 to p15 l54
**Units of study** - Number and relevant characteristics of participants, documents, or events included in the study; level of participation (could be reported in results)	n/a
**Data processing** - Methods for processing data prior to and during analysis, including transcription, data entry, data management and security, verification of data integrity, data coding, and anonymization/de-identification of excerpts	p16/l24 to p17/l40
**Data analysis** - Process by which inferences, themes, etc., were identified and developed, including the researchers involved in data analysis; usually references a specific paradigm or approach; rationale**	p16/l24 to p17/l40
**Techniques to enhance trustworthiness** - Techniques to enhance trustworthiness and credibility of data analysis (e.g., member checking, audit trail, triangulation); rationale**	p17/l10-40

Results/findings

**Synthesis and interpretation** - Main findings (e.g., interpretations, inferences, and themes); might include development of a theory or model, or integration with prior research or theory	n/a
**Links to empirical data** - Evidence (e.g., quotes, field notes, text excerpts, photographs) to substantiate analytic findings	n/a

Discussion

**Integration with prior work, implications, transferability, and contribution(s) to the field** - Short summary of main findings; explanation of how findings and conclusions connect to, support, elaborate on, or challenge conclusions of earlier scholarship; discussion of scope of application/generalizability; identification of unique contribution(s) to scholarship in a discipline or field	n/a
**Limitations** - Trustworthiness and limitations of findings	n/a

Other

**Conflicts of interest** - Potential sources of influence or perceived influence on study conduct and conclusions; how these were managed	p22/l24-28
**Funding** - Sources of funding and other support; role of funders in data collection, interpretation, and reporting	p22/l10-20

*The authors created the SRQR by searching the literature to identify guidelines, reporting standards, and critical appraisal criteria for qualitative research; reviewing the reference lists of retrieved sources; and contacting experts to gain feedback. The SRQR aims to improve the transparency of all aspects of qualitative research by providing clear standards for reporting qualitative research.

For peer review only - http://bmjopen.bmj.com/site/about/guidelines.xhtml

**The rationale should briefly discuss the justification for choosing that theory, approach, method, or technique rather than other options available, the assumptions and limitations implicit in those choices, and how those choices influence study conclusions and transferability. As appropriate, the rationale for several items might be discussed together.

Reference:
O'Brien BC, Harris IB, Beckman TJ, Reed DA, Cook DA. **Standards for reporting qualitative research: a synthesis of recommendations.** *Academic Medicine*, Vol. 89, No. 9 / Sept 2014 DOI: 10.1097/ACM.0000000000000388

For peer review only - http://bmjopen.bmj.com/site/about/guidelines.xhtml
